# Comparative in vitro toxicity of compositionally distinct thermal spray particulates in human bronchial cells^☆^

**DOI:** 10.1016/j.toxrep.2024.101851

**Published:** 2024-12-04

**Authors:** E.S. Burns, R.E. Harner, V. Kodali, A.A. Afshari, J.M. Antonini, S.S. Leonard

**Affiliations:** a Health Effects Laboratory Division, National Institute for Occupational Safety and Health, Morgantown, WV, United States; b West Virginia University, School of Medicine, Morgantown, WV, United States

**Keywords:** Thermal spray, In vitro, Inhalation toxicology, Electric arc, Occupational health

## Abstract

Thermal spray, in general, is a process that involves forcing a melted substance, such as metal or ceramic in the form of wire or powder, onto the surface of a targeted object to enhance its desired surface properties. In this paper, the melted substance is metal wire generated by an electric arc and forcibly coated on a rotary iron substrate using compressed air. This thermal process is referred to as double-wire arc thermal spray. The particles generated through these methods fall within the nanometer to micrometer agglomerate size range. There is concern regarding potential human health outcomes as these particles exhibit a similarity in particle morphology to welding fumes. Thermal spray wires with zinc (PMET540), iron and chromium (PMET731), and nickel (PMET885) as primary metal compositions were used to generate particulate via an electric arc wire thermal spray generator for exposure to human bronchial cells (BEAS-2B) to examine comparative toxicity ranging from 0 to 200 µg/mL. Resulting cellular viability was assessed through live cell counts, and percent cytotoxicity was measured as a function of LDH release. Oxidative stress, genotoxicity, and alteration in total antioxidant capacity were evaluated through DNA damage (COMET analysis) and antioxidant concentration at 0, 3.125, 25, and 100 µg/mL. Protein markers for endothelin-1 (ET-1), interleukin-6 (IL-6), and interleukin-8 (IL-8) were also assessed to determine inflammation and endothelial alteration.

**Results:**

indicate modulation of oxidative stress response in a material and dose dependent manner. PMET540 exhibited the greatest cytotoxic effect between wires and across doses. DNA damage and antioxidant concentration induced by PMET540 were significantly higher than other wires at higher doses (DNA damage increased at 25 and 100 µg/mL; Antioxidant concentration increased at 100 µg/mL). However, ET-1 concentration significantly increased only after application of 100 µg/mL PMET885. IL-6 and IL-8 were most highly expressed in BEAS2B culture after 25 µg/mL exposure to PMET540 (99.4 % Zn). This data suggests that metal composition of thermal spray wires dictates the diverse response in human bronchial cells.

## Highlights

• Zinc-based thermal spray particles (PMET540) induced the highest level of cytotoxicity and DNA damage in bronchial cells.
• Increased Endothelin-1 production was significant in bronchial cells exposed to Nickel-based thermal spray (PMET885).
• Comparative trend in cytotoxicity differs between submerged *in vitro* cell culture data and *in vivo* results.

## Graphical Abstract

**Figure:**
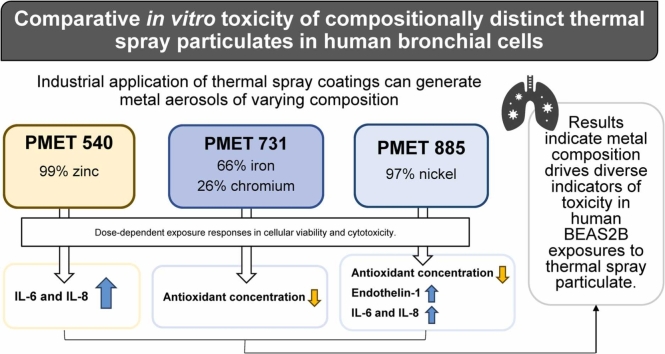

## 1 Introduction

Metal thermal spray is a prevalent technology utilized to deposit coatings on surfaces for the purpose of improving conductivity, protecting materials from rust or oxidation, refinishing materials, and related purposes. Thermal spray wires can vary greatly in metal composition. Common elemental metals present in thermal spray wires include zinc (Zn), copper (Cu), chromium (Cr), nickel (Ni), cobalt (Co), manganese (Mn), and iron (Fe). Inhalation of these metals has been associated with respiratory irritation, pulmonary cancers, and metal fume fever (as reviewed by [5]). The particle morphology of thermal sprays has been observed to be similar to that produced from welding fumes under scanning electron microscopy [5] as both form long chains or agglomerates of metal particulate. This similarity raises concerns about outcomes of thermal spray inhalation as welding fumes have been classified as carcinogenic to humans (Group 1) by the International Agency for Research on Cancer [25] and are known to confer risk of lung disease [26].

Beyond similar particle morphology, it is of note that welding fumes often contain similar elemental metals as can be found across thermal spray wires (such as Mn, Cr, Fe, Ni, etc.) although composition varies by material and application method (i.e., mild steel versus stainless steel welding) [32], [16]. Worker health studies have reported a higher occurrence of occupational asthma in welders compared to workers in other job descriptions within the same companies [20], and welding-associated asthma has specifically been correlated with chromium exposure [9]. Concerningly, soluble portions of welding fume nanoparticulates can also translocate via vasculature to other organs after inhalation [19], [4]. This concern regarding similar health impacts from thermal spray inhalation exposures may be supported by occupational observations in thermal spray facilities – elevated metal levels have been found in the urine of workers after thermal spray administration [10].

In extreme cases, occupational thermal spray exposure can even result in death if personal protective equipment is not applied [34]. Unpublished industrial hygiene reports from thermal spray coating facilities have indicated exposures from 20 to 100 times the OSHA permissible exposure limit for hexavalent chromium (5 µg/m^3^) [5] suggesting that workers may still be at risk of metal inhalation during thermal spray processes despite implementation of engineering controls. Given the pathologic outcomes for hexavalent chromium described in welding-associated studies [33], [9], thermal spray-related inhalation poses a likely risk for development of respiratory disease. Furthermore, inhalation of elemental metals such as Ni and Cr have been associated with development of lung cancers, whereas Zn inhalation may lead to metal fume fever – all of which are commonly found within various thermal spray wires [5].

To examine the comparative in vitro toxicity between wires of different majority composition, wires with majority Zn (PMET540; 99.4 % Zn, 0.325 % Ni, and 0.235 % Fe), stainless steel (PMET731; 66.3 % Fe, 26.2 % Cr, 1.02 % Mn), and nickel (PMET885; 96.9 % Ni, 1.69 % Al, 1.06 % Zn, 0.699 % Fe) compositions were used to generate thermal spray particles in an onsite electric arc wire thermal spray generator [1] for exposure to human bronchial cells (BEAS-2B) in submerged culture. Metrics of toxicity were assessed through live cell counts, lactate dehydrogenase (LDH) activity, DNA damage as measured by COMET analysis, total antioxidant capacity of cells, endothelin-1 (ET-1) concentration, as well as interleukin-6 (IL-6) and interleukin-8 (IL-8) production to capture distinct markers of immune and pro-inflammatory response. Data indicates differential cellular response between materials and doses, suggesting that metal composition is a driving factor in bronchial cell response across distinct thermal spray wires.

## 2 Methods

### 2.1 Cell culture

BEAS-2B cells were grown and maintained in cell culture flasks at 37 °C with 5 % CO_2_. Culture medium (BEGM, Lonza) was changed three times per week throughout the culturing procedure. The evening prior to exposure, cells were seeded overnight at 0.55 × 10^5^ cells/mL (Passage 6) into individual wells of 96- or 6-well plates.

### 2.2 Thermal spray exposures

Thermal spray fumes were generated by an automated electric arc wire thermal spray generator as previously described for the characterized PMET540, PMET731, and PMET885 wires [1], [17]. Thermal spray particles for each rod type were weighed into aliquots for short-term storage and consequent preparation of stock solutions. Particles were then resuspended in 0.6 mg/mL bovine serum albumin (BSA) in PBS to a stock concentration of 5 mg/mL. To prevent aggregation, stock solutions were prepared from dry aliquots, sonicated (Branson Sonifier 450 with Cup Horn in Sound Enclosure) at 70 % amplitude for 30 seconds, and immediately serially diluted in BEGM cell culture medium to test conditions.

For survival and LDH curves, doses were prepared at 200, 100, 50, 25, 12.5, 6.25, 3.125, 1.56, and 0 µg/mL to complement macrophage toxicity data described by Kodali et al. [17]. For the subsequent exposures, three doses were selected from the toxicity curves to represent a low, medium, and high dose based on LDH response and survival data. Concentrations of 100, 25, 3.125, and 0 µg/mL were chosen due to the propensity to capture a range of cellular responses. At 100 µg/mL, statistical analysis showed significant differences in toxicity. While there was no significant difference from controls at 25 and 3.125 µg/mL, lower doses were selected to illustrate potential acute changes in protein secretion prior to significant cell death.

For the two assays (Viability and LDH) that utilized the complete dose curve from 200 to 0 µg/mL for each tested wire material, all exposures were conducted in individual 96-well plates for each material (PMET 540, 731, and 885). Each 96-well plate was seeded with four independent biological replicates per dose condition (n = 4) from BEAS-2B cells grown and passaged in separate culture flasks. A negative control or 0 µg/mL dose was present for each biological replicate across each thermal spray material plate exposure and consisted of culture medium and 0.6 mg/mL BSA. For the LDH assay, additional wells were seeded at the same concentration in order to provide unexposed culture wells for the incubation of high and low controls as required by the test protocol (see *Cytotoxicity* section below).

For the remaining assays that were conducted at 0, 3.125, 25, and 100 µg/mL, negative controls (0 µg/mL) were pooled as a test condition (n = 3) rather than conducted repeatedly for each individual material exposure as above. As such, these points are represented graphically as a control rather than 0 µg/mL, although both labels are functionally identical as each seeded well only received 0.6 mg/mL BSA in culture medium for both condition labels during the exposure series. For these assays, three biological replicates (n = 3) were also conducted per wire material across exposure conditions, with three technical replicates applied per test to discern intra-assay variability. Technical replicates were averaged prior to statistical analysis of results. All exposure series were conducted in 6-well plates at the same seeding density as before (0.55 ×10^5^ cells/mL).

Immediately before exposure, cell culture medium was aspirated from BEAS-2B culture plate wells, cells rinsed with 1x PBS (Ca and Mg free), and the relevant dose administered to individually cultured biological replicates. Cells were then exposed under incubation conditions for 24-hours before endpoint analysis. Post-exposure, culture medium was removed for total antioxidant capacity and LDH release assessments as well as ET-1, IL-6, and IL-8 ELISA assays. Cultured cells were used for cell counts and COMET analyses.

### 2.3 Viability

Cell counts were conducted using the PromoCell Detach Kit (PromoCell, Cat. # C-41210), trypan blue, and a Countess II automated cell counter (Invitrogen) to assess total estimated cells/mL and total live cells/mL. Four separate cell suspension samples across four Countess wells were used to generate estimate live cell concentrations (n = 4) in a single technical replicate each as limited by the viewable slide area. If all cells were deceased in a viewable slide well, this was denoted as 0 cells/mL and included in the results. In the event that cell/mL were unreadable by the Countess II due to large, undifferentiable cell aggregates, this observation was not recorded.

### 2.4 Cytotoxicity

Lactate dehydrogenase release into culture medium was used as a proxy for cell membrane damage (Sigma Aldrich, Cat. # 11 644 793 001). LDH-dependent reduction of NAD+ transferred H+ to tetrazolium salts, producing formazan salts in solution and causing a colorimetric shift in response. Percent cytotoxicity values were calculated from absorbance readings of experimental samples and controls (Cytotoxicity Detection Kit, Roche). Colorimetric LDH tests were conducted across four biological replicates per condition (PMET540, PMET731, PMET885 applied at 0, 3.125, 25, and 100 µg/mL) and were complemented with four technical replicates for high controls by dose (Triton X-100 administered post-exposure). High controls represented the colorimetric response produced by BEAS-2B cultures at the defined seeding density (0.55 ×10^5^ cells/mL) at approximately 100 % cytotoxicity and were used as a correction factor in percent cytotoxicity calculations as directed by Roche.

### 2.5 Comet assay

Comet electrophoresis was used to draw charged DNA fragments through porous agarose medium to create an observable “tail” of DNA when imaged under fluorescent filters. Three biological replicates were exposed to 0, 3.125, 25, or 100 µg/mL per each thermal spray material (n = 3). After the 24-hour exposure, cells in independent treatment wells were rinsed with PBS and detached according to the PromoCell Detach Kit protocol with volumes adjusted for surface area of 6-well plate wells (Corning). Detached cells were diluted and added to agarose on glass slides as described by the manufacturer’s protocol (Trevigen); two glass slide wells were prepared per each biological sample (two technical replicates per condition). Double-stranded DNA was stained with SYBR green (Invitrogen, Cat. #S-7585).

Images were taken at random locations across each slide using an Olympus BX63 Fluorescent Microscope and cellSens imaging software (Olympus). All individual cell COMETs were measured within each image using Comet Assay IV software (Perceptive Instruments). Data was not taken if comet overlap precluded tail measurement or if a cell was truncated by the image bounds. Tail moment, calculated by taking the comet tail length times the percent DNA within the tail, was used a metric for the average extent of DNA damage caused by each treatment condition and was automatically scored by the Comet Assay IV suite.

### 2.6 Total antioxidant capacity

Antioxidant concentration in culture medium was assessed using the Total Antioxidant Capacity Assay Kit (Sigma Aldrich, Cat. # MAK187) according to manufacturer’s protocols across three biological replicates (n = 3), each of which were divided into three technical replicates to evaluate assay variability, per condition (PMET540, PMET731, PMET885 applied at 0, 3.125, 25, and 100 µg/mL). Cellular production of non-enzymatic antioxidants was measured by protein and small molecule antioxidant reduction of Cu^2+^ by Cu^+^ chelation of colorimetric probes. Absorbance values were read at 570 nm (BioTek Synergy H1 Plate Reader, Agilent Technologies) and antioxidant concentration calculated versus known values generated from the Trolox standard curve.

### 2.7 Endothelin-1

Endothelin-1 ELISA (Abcam, Cat. # ab133030) was conducted according to the manufacturer’s protocol. Culture fluid was assessed for secreted ET-1 concentration after 4- and 24-hours post-exposure recovery periods across three biological and three technical replicates per condition (PMET540, PMET731, PMET885 applied at 0, 3.125, 25, and 100 µg/mL). Absorbance was read at 450 nm (BioTek Synergy H1 Plate Reader, Agilent Technologies) immediately after the addition of stop solution. Absorbance values were blank corrected before calculating protein concentration (pg/mL) values from the standard curve.

### 2.8 Interleukin-6 and Interleukin-8

Modulation of IL-6 and IL-8 expression was assessed through ELISA quantification of protein concentration to examine acute immune and inflammatory response in BEAS-2B culture. Interleukin ELISA assays were conducted using Invitrogen Human IL-6 and IL-8 ELISA kits according to the manufacturer’s protocol (ThermoFisher Scientific, Cat. # KHC0061 & KHC0081). All ELISA tests were conducted across three biological replicates per each material (PMET540, PMET731, PMET885) and dose condition (0, 3.125, 25, or 100 µg/mL) with each single biological replicate divided into three technical replicates per assay. Incubation steps were carried out in the dark at room temperature. Chromogenic stop solution was added immediately before the plate absorbance values were read at 450 nm (BioTek Synergy H1 Plate Reader, Agilent Technologies). All samples were blank corrected for background signal prior to concentration calculations form the standard curve.

### 2.9 Statistics

All statistical analyses were performed in R using RStudio (ver. 2024.04.1, Posit Software, PBC). Normality and distribution of data were assessed by a Shapiro test and Q-Q plot prior to statistical analysis. Two-way analysis of variance (ANOVA) and Tukey’s Honestly Significant Differences (HSD) tests were applied to determine significance and parameter interactions by dose and distinct thermal spray composition. Statistical significance was considered at p < 0.05.

## 3 Results

### 3.1 Thermal spray particle characteristics

Particle characterization, including mass mean aerodynamic diameter MMAD) and the geometric standard deviation (GSD), of PMET540, PMET731, and PMET885 wires (Table 1) were reported by Afshari, et al. [1] and Antonini, et al. [6]. The resultant elemental composition of thermal spray particulate from each wire was confirmed by scanning electron microscopy. The collected PMET540 particulate was composed of 99.4 % Zn, 0.325 % Ni, and 0.235 % Fe. Stainless steel PMET731 wire particles were found to contain 66.3 % Fe, 26.2 % Cr, and 1.02 % Mn. Particulates generated from PMET885 steel contained 96.9 % Ni, 1.69 % Al, 1.06 % Zn, and 0.699 % Fe. The MMAD (310–378 nm), GSD (1.89–1.94), and surface area (9.15 ×10^5^ – 1.12 ×10^6^ µm^2^/cm^3^) of generated particles remained similar across each wire type [1], [6].

**Table 1:** Particle characteristics of thermal spray wires as assessed by gravimetric measurements and electron scanning microscopy.

Thermal Spray Wire	Elemental Composition (by percent weight)	Mean Mass Aerodynamic Diameter (MMAD)	Geometric Standard Deviation (GSD)	Surface Area
PMET540	99.4 % Zn ( ± 0.24) 0.325 % Ni ( ± 0.16) 0.235 % Fe ( ± 0.14)	378 nm	1.89	9.15 × 10^5^ µm^2^/cm^3^
PMET731	66.3 % Fe ( ± 0.56) 26.2 % Cr ( ± 0.57) 1.02 % Mn ( ± 0.02)	310 nm	1.94	1.03 × 10^6^ µm^2^/cm^3^
PMET885	96.9 % Ni ( ± 0.63) 1.69 % Al ( ± 0.03) 1.06 % Zn ( ± 0.32) 0.699 % Fe ( ± 0.11)	316 nm	1.94	1.12 × 10^6^ µm^2^/cm^3^

Table data as modified from Afshari, et al. [1] and Antonini, et al. [6].

### 3.2 Cell viability

**Fig. 1:**
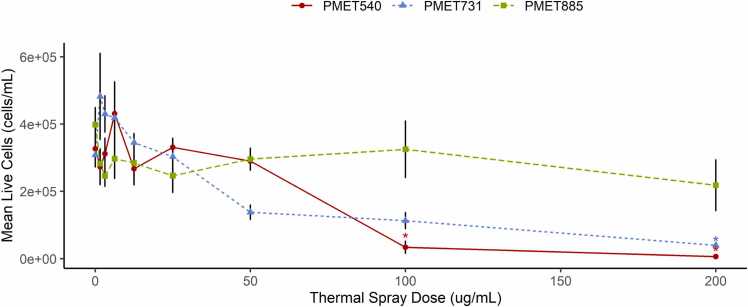
Viability as described by mean live cells (cells/mL) across dose and thermal spray wires. Average live cell concentration decreased significantly for PMET540 treatment at 100 µg/mL and 200 µg/mL and for PMET731 at 200 µg/mL when compared to viability at lower doses. Cell counts did not differ significantly across PMET885 exposures. Error bars correspond to standard error. Significance is represented by an asterisk (*; p < 0.05) when a treatment differed from the control. See Table S1 for full significance groups across material and dose as assigned by two-way ANOVA and Tukey’s HSD post hoc analyses.

Cell counts were used to examine live cells after exposure. The resulting viability trend showed a general decrease in cells/mL as dose increased for all thermal wires (Fig. 1). A significant difference in remaining live cells was observed between wire types only at the 100 µg/mL dosage; application of PMET885 and PMET540 caused a significant difference in BEAS2B culture viability between wires. However, dose-dependent changes in viability also occurred within treatments. Live cells decreased significantly between 100 and 200 µg/mL PMET731 and 1.56–6.25 µg/mL of PMET731. Similarly, the total live cells observed at 100 and 200 µg/mL PMET540 decreased significantly from the values observed from 0, 6.25, and 25 µg/mL PMET540. Despite trends in overall viability, cell count did not differ significantly across PMET 885 application.

### 3.3 Cytotoxicity

**Fig. 2:**
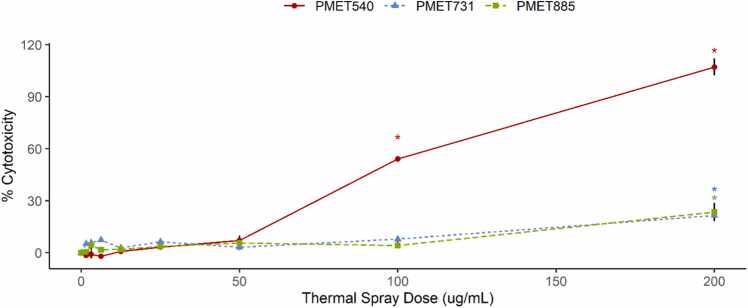
Lactate dehydrogenase (LDH) release as an indicator of cell membrane damage. LDH release from damaged cell membranes and thus inferred cytotoxicity increased significantly (p < 0.05) at 100 and 200 µg/mL PMET 540 and at 200 µg/mL for PMET731 and PMET885. Significance labels (*; p < 0.05) represent values significantly different from both the control and other unlabeled points. Error bars represent standard error.

To examine cytotoxic effect of thermal wire application, release of LDH into culture medium was used as a metabolic indicator for cell membrane integrity. Exposure to PMET731 and PMET885 produced an increase in percent cytotoxicity at the highest dose (200 µg/mL) (Fig. 2). PMET540 increased LDH release significantly beginning at 100 µg/mL and again at 200 µg/mL both by dose and compared to other thermal spray wires at each exposure concentration (Fig. 2).

### 3.4 CometAssay shows DNA damage dose response

**Fig. 3:**
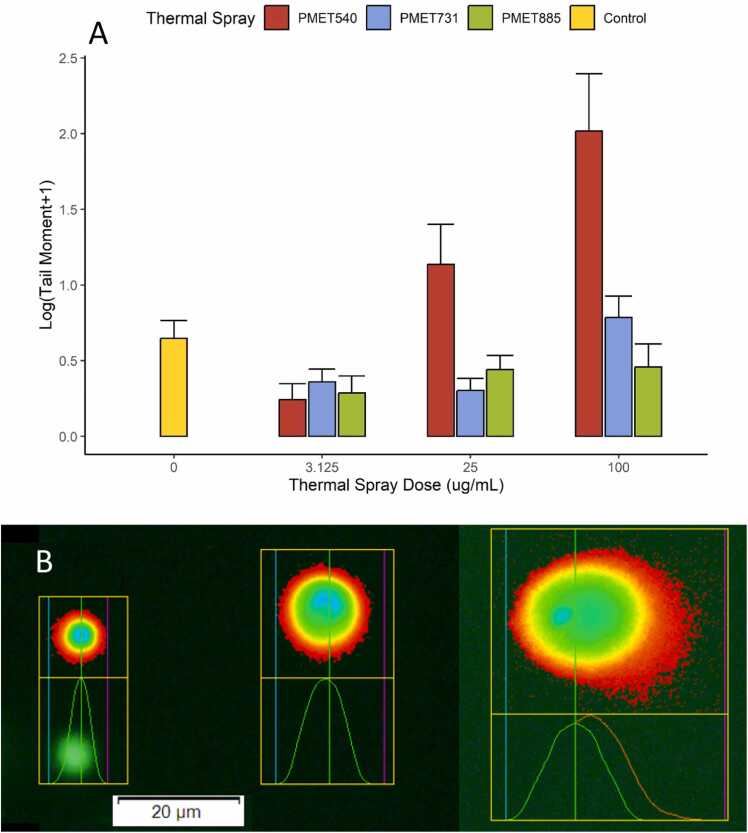
DNA damage induced by PMET exposure in BEAS-2B cells. A) Tail moment (tail length x % DNA in tails) is used as a measure of total DNA damage. PMET540 exposure significantly increased DNA damage from 3.125 μg/mL to 25 and 100 μg/mL treatments, though these values did not differ significantly from the control. Error bars represent standard error. B) Representative Comets from 3.125, 25, and 100 µg/mL of PMET540 exposures, respectively.

DNA damage from thermal spray exposure was assessed through the comet assay using SYBR green to stain double-stranded DNA in individual BEAS-2B cells post-treatment. Mean tail moment, and thus observed DNA damage, differed significantly between wires at 25 µg/mL and between PMET540 and PMET885 at 100 µg/mL (Fig. 3A). While treatments did not significantly differ from the control, tail moment increased significantly between application of PMET540 at 3.125 µg/mL and both 25 and 100 µg/mL. Representative images of BEAS-2B comets are shown in Fig. 3B and illustrate various observed tail lengths on comets used for analysis (representative images taken from PMET540 treated cells across different treatments).

### 3.5 Total antioxidant capacity

**Fig. 4:**
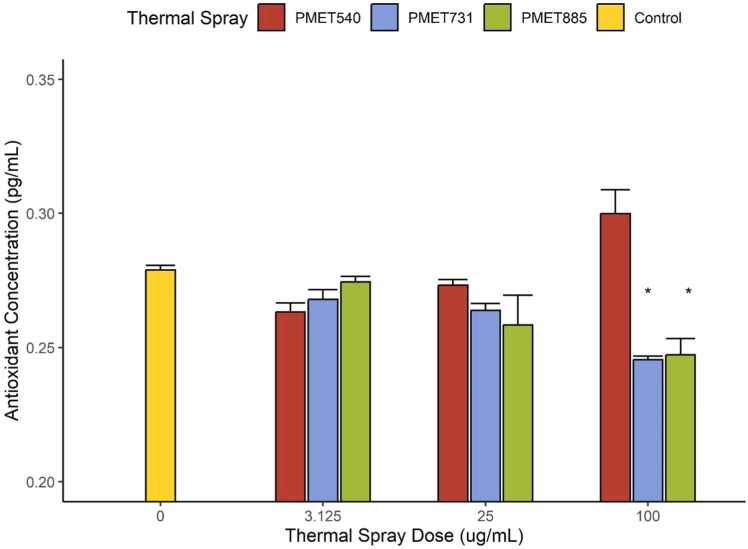
Total Antioxidant Capacity of cells as measured by non-enzymatic antioxidant conversion of copper probes. Oxidative stress causes consumption of cellular antioxidants to combat oxidative damage but may also upregulate antioxidant production. Non-enzymatic protein and small molecule antioxidants present after exposures reduced copper probes to produce a colorimetric response comparable to a standard curve of known antioxidant concentrations. Thermal spray exposure significantly reduced BEAS2B antioxidant concentration at 100 µg/mL PMET731 and PMET885. While not significant from the control, the increase in antioxidant concentration did change significantly between 25 and 100 µg/mL of PMET540 exposure. Significance is represented by an asterisk (*; p < 0.05) when a treatment differed from the control. Error bars represent standard error.

Between thermal spray rods, only PMET540 induced significant increase in antioxidant concentration compared to lower doses when exposed at 100 µg/mL (Fig. 4). Conversely, at 100 µg/mL, PMET731 and PMET885 significantly decreased antioxidant concentration within BEAS2B cultures when compared to the controls (0 µg/mL).

### 3.6 Endothelin-1

**Fig. 5:**
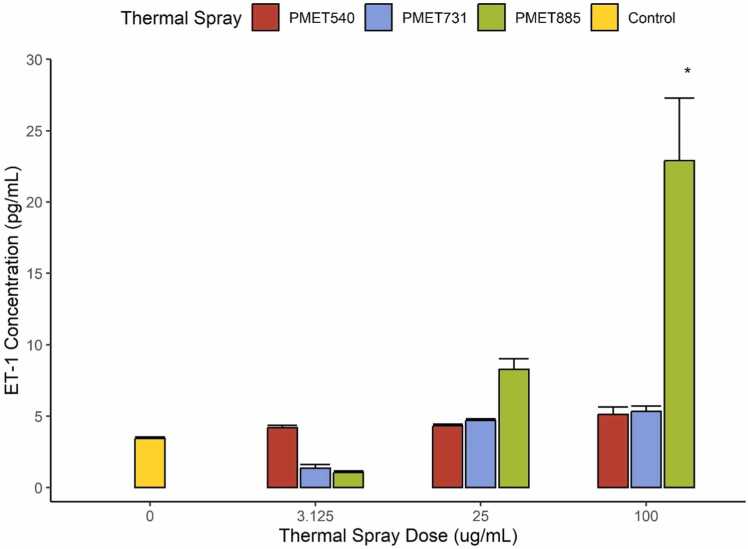
Endothelin-1 Release from BEAS-2B cells after thermal spray exposure. A significant increase in ET-1 release was observed after 100 µg/mL PMET885 exposures. Significance is represented by an asterisk (*; p < 0.05) when a treatment differed from the control. Error bars represent standard error.

ET-1 production in human bronchial cells plays a role in the induction of bronchoconstriction responses and progression of multiple airway pathologies [15]. BEAS2B cells produced an increase in ET-1 protein concentrations at 100 µg/mL exposures of PMET885 (Fig. 5). At 25 µg/mL PMET885, there was a significant increase in ET-1 response compared to that of 3.125 µg/mL but this increase in ET-1 concentration was not significantly different when compared to the control.

### 3.7 IL-6 and IL-8

**Fig. 6:**
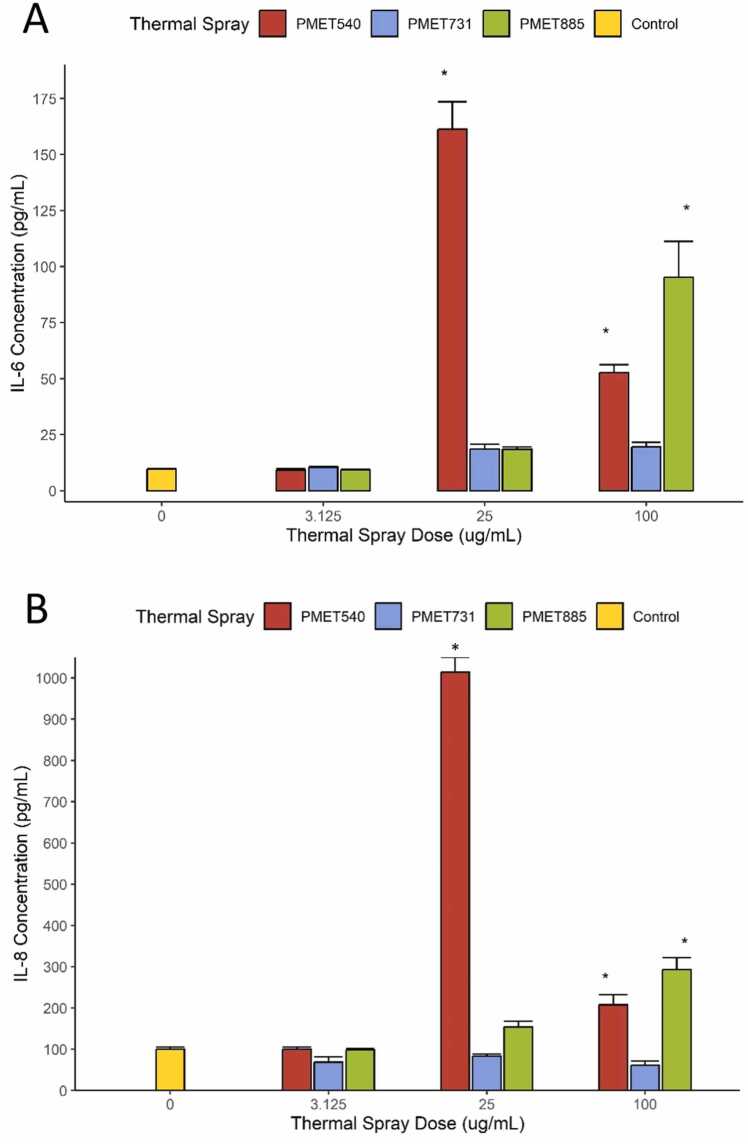
Interleukin response (IL-6 and IL-8) of BEAS-2B cells after thermal spray exposure. A) IL-6 and B) IL-8 production increased significantly in cells after 25 µg/mL application of PMET 540. In thermal spray exposures at the 100 µg/mL concentrations, PMET7885 and PMET540 increased IL-6 (A) and IL-8 (B) concentration significantly compared to both PMET731 and the controls. Significance is represented by an asterisk (*; p < 0.05) when a treatment differed from the control. Error bars represent standard error.

A significant increase in IL-6 and IL-8 occurred for both PMET450 and PMET885 at higher doses. BEAS2B cells produced a significantly elevated amount of both IL-6 and IL-8 at 25 µg/mL and 100 µg/mL PMET540, although total concentration of both interleukins was reduced at 100 µg/mL from the 25 µg/mL dose (Fig. 6A&B). At 25 µg/mL, PMET540 produced the highest measured interleukin concentration (in both IL-6 and IL-8) compared to any other dose or wire. Exposure to PMET885 at 100 µg/mL produced a significant increase in both IL-6 and IL-8 concentrations from both the previous PMET885 dose and when compared to the other thermal spray wires of the same dose.

## 4 Discussion

Due to the potential severity of metal-induced pulmonary disease, many studies have aimed to interpret the contribution of different elemental metals or metal fractions to lung inflammation and immune response. Ni and Cr are known human carcinogens that contribute to the development of other lung pathologies such as asthma and chronic obstructive pulmonary disease (COPD) [7], [12], [24], [27]. Furthermore, transition metals such as Fe, Ni, and Cr, can generate free radicals and have been shown to do so after exposure to stainless steel welding fumes [19], [24], [3]. Particle characterization of PMET540, PMET731, and PMET885 conducted by Antonini, et al. [6] included electron paramagnetic resonance (EPR) analysis of hydroxyl radicals which showed the highest levels of surface reactivity occurring within PMET540 and PMET731 samples. Inhalation of particulate capable of generating free radicals can disrupt cell membranes, damage organic macromolecules (i.e., nucleotides), and impair phagocytosis, ultimately leading to cellular damage, cytokine release, and even cell death [13], [31]. To better understand the potential intersection of material reactivity and metal-induced inflammation and immune response, DNA Damage and total antioxidant capacity were assessed as both a result of and metabolic compensation for oxidative stress, respectively. As cytokine release has functional roles across inflammatory response, immune modulation, and oxidative stress response, interleukin response can be considered both independently and in conjunction with oxidative stress. Due to the pleiotropic effect of ET-1 and its associations with multiple respiratory pathologies, increased production of this protein was considered an indicator of potential adverse pulmonary outcomes that may not be discretely linked to reactive potential of the applied materials.

Within our study, PMET540 (containing 99 % Zn) caused the most significant changes between all materials. PMET540 elicited a significant dose-dependent decrease in cell count across treatments and cytotoxicity became significantly elevated after 100 µg/mL exposures. Exposure of BEAS-2B cultures to PMET540 also produced an increase in antioxidant concentration at higher doses (25 and 100 µg/mL, Fig. 4). These elevated values suggest that bronchial cells may upregulate antioxidant production as PMET540 dose increases to compensate for the impact of oxidative stress or may be further elevated from intracellular leakage of small molecule antioxidants due to increased cellular damage that was observed at these higher doses. Evidence for the induction of oxidative stress by dose is supported by DNA damage observed in the comet analyses and increasing cytotoxicity as measured by LDH production. DNA fragmentation, as estimated by tail moment scoring, increased after 3.125 µg/mL. Similarly, BEAS-2B production of IL-6 and IL-8 was significant at 25 and 100 µg/mL PMET540, but total protein concentration fell at the 100 µg/mL dose. This decrease in interleukin concentration likely corresponds to the cytotoxicity values and total live cells indicated at this dose. At a 100 µg/mL PMET540, LDH derived cytotoxicity in BEAS-2B was elevated to 54 %, and an average of 10.3 % of cells remained viable in culture compared to the negative control. IL-6 is associated with acute phase inflammatory response, including recruitment of macrophages and lymphocytes; while it can induce proinflammatory as well as anti-inflammatory signaling, it is generally considered a marker of post-injury inflammation regardless of downstream function [2], [30]. IL-8 is most often associated with immune response and the chemotactic recruitment and infiltration of neutrophils to mediate pulmonary inflammation [14], [18], [29]. A significant response in both these factors suggests multiple types of inflammatory response are upregulated in response to PMET540 exposures.

Notably, live cell counts did not differ significantly from the control across PMET885 dose. Despite this result, exposure to PMET885 (93 % Ni, 5.3 % Al) showed significant DNA damage and percent cytotoxicity at the highest measured doses (100 µg/mL and 200 µg/mL, respectively). PMET885 also produced a significant increase in interleukin concentration at 100 µg/mL exposures, although the impact of PMET540 was more pronounced across both wire types and dose. Unique to PMET885 alone was the significant production of ET-1 at 100 µg/mL exposures. Release of proinflammatory cytokines is known to prompt expression of ET-1 in normal human bronchial epithelia, as well as in adjacent pulmonary tissues [15]. ET-1 elevation at high doses suggests the potential for bronchoconstriction or other ET-1 mediated effects in response to high levels of exposure to nickel-based rods. ET-1 elevation has been implicated in asthma, COPD, and pulmonary fibrosis pathogenesis [11], [15], [22], [28]. Several publications and case studies have linked occupational asthma to nickel exposures and sensitivities [12], [21], [23], [8]; however, the diversity of mechanisms in both ET-1 production and action makes it difficult to predict pathologic outcomes. These findings, however, suggest an association between the development of nickel-based pathologies from other occupational hazards and the potential for Ni-reactivity in response to thermal spray generated from wires such as PMET885.

Particles generated from the stainless steel rod, PMET731 (69.8 % Fe, 23.5 % Cr, 5.3 % Al, 0.45 % Mn), induced no significant responses in either ET-1, IL-6, or IL-8 production in BEAS-2B cells across all exposure doses when compared to the control. The trend in cytotoxicity response is comparable by dose to those observed in PMET885; however, at the highest concentrations (100 and 200 µg/mL) of PMET731 live cell numbers decreased significantly from lower doses (i.e., 1.56–12.5 µg/mL). This suggests that exposure to PMET731 still induces cytotoxicity at high concentrations (200 µg/mL) but does not elicit the same inflammatory response mechanisms observed with PMET885 and PMET540 at equivalent doses.

The responses observed in our PMET731 exposures is congruent with findings of other studies where, *in vitro*, stainless steel rod PMET720 (82.2 % Fe, 13.4 % Cr, 2.37 % Mn, 1.31 % Zn) only induced cytotoxicity and damage to cellular membranes of RAW 264.7 macrophages at the highest dose (200 µg/mL) [17]. However, *in vivo* inhalation exposures in rats demonstrated modulation of cytokine and chemokine production as measured in bronchioalveolar lavage fluids (BALF), suggesting that cytokines other than IL-6 and IL-8 may be integral in pulmonary response to stainless steel thermal sprays [17].

*In vitro* cell culture methodologies are increasingly employed as a screening method for toxicology and human health studies as well as a mechanism to reduce burden in *in vivo* research applications. Interestingly, when compared to the results of the current *in vitro* study, a previous *in vivo* inhalation study of the same thermal spray materials (PMET540, PMET731 and PMET885) by Antonini, et al. [6] describes a contrasting pattern in material toxicity. Evaluation of BALF collected from male Sprague-Dawley rats at 4- and 30-days post-exposure showed a significant and sustained LDH release from PMET885 exposure when compared to both controls and other thermal spray aerosols (compared as % air control), with PMET885 toxicity further supported by cell proliferation and lung deposition data [6]. It is, however, noted that respiratory clearance of PMET540 proceeded more quickly compared to PMET731 and PMET885. In the current study, without the assistance of mucociliary clearance inherent to the respiratory tract, the soluble metal fraction of PMET540 particulate is retained along with the insoluble fractions for the full duration of *in vitro* exposures. When compared to EPR spectrographs [6], the results provided by these BEAS-2B submerged cell culture exposures more closely agree with the reactive potential of their material components.

This assessment of PMET540, PMET731, and PMET885 was conducted in submerged human bronchial cells to compare biological trends with existing macrophage and *in vivo* data on thermal spray exposures. The doses used in this study were intended to capture a broad range of toxicological response as human occupational exposures can vary greatly from industry, location, process, and the applied engineering controls [6]. These data indicate PMET540 induces the highest levels of cytotoxicity in human bronchial BEAS-2B cell culture and elicits a significant response at lower doses than the other tested materials. However, the results also suggest that there was no single common type of toxicity and, rather, each wire may produce disparate effects in human bronchial cells. DNA fragmentation observed in the comet assay along with elevated antioxidant production in response to PMET540 exposures provide some evidence for the capacity to induce oxidative stress in human bronchial cells, although the source of antioxidant increase is not conclusive as it was assessed from culture medium rather than lysate. However, the induction of ET-1 by PMET885 seems to suggest that elemental composition may impact the severity of cytokine or potential bronchoconstrictive responses after inhalation. When these present data are considered in the light of a feasibility study, it is clear that submerged *in vitro* culture is adequate in discerning material-driven differences in human proinflammatory and immune response but may not be an adequate tool alone for anticipating health impacts of human exposure due to physical limitations and simplicity of the culture and exposure methods themselves. One such benefit to human cell line *in vitro* exposures is the incorporation of human cell metabolism in endpoint assessment as murine models can differ from humans in both airway physiology and gene homology. As highlighted by comparative studies, a holistic approach in considering composition, solubility, and clearance through application of both models may improve the accuracy with which we anticipate human health outcomes.

Overall, the observed responses in this study suggest the unique metal components in each wire likely impact the pulmonary system through independent pathways. Additional studies on the impacts of metal composition through different exposure models, including evaluations of material solubility and a larger panel of cytokine and chemokine response, may help elucidate altered bronchial response. Future studies will extrapolate these findings to exposures using human bronchial cells cultured at the Air Liquid Interface (ALI) and a wider assessment of pro-inflammatory markers to better describe the contribution of metal composition to respiratory response in human cells and the different immune or inflammatory pathways which these materials may elicit. Follow-on work will seek to address whether increased physiological complexity of *in vitro* models, such as the differentiated epithelia generated by ALI, can recover the trends in toxicity observed in whole-lung *in vivo* exposure studies to further validate and provide greater context for *in vitro* material toxicity screening practices.

## Declaration of Competing Interest

The authors declare that they have no known competing financial interests or personal relationships that could have appeared to influence the work reported in this paper.

## Data Availability

Data will be made available on request.
