# Supplementary material for: Comparative in vitro toxicity of compositionally distinct thermal spray particulates in human bronchial cells
Source: Toxicol Rep. 2024 Dec 4;13:101851. doi: 10.1016/j.toxrep.2024.101851 (PMC11665665; doi:10.1016/j.toxrep.2024.101851)
Supplement: Supplementary file 1 — Supplementary material [file mmc1.docx]

**Supplemental Table 1**. Mean live cell concentrations (represented as 10^4^ cells/mL) and significance groups (p < 0.05) by dose and wire as assigned by ANOVA and Tukey’s HSD post hoc analysis. Significance labels correspond to alphabetical groups assigned by two-way ANOVA and Tukey’s HSD post hoc analyses; values are significantly different if group designations do not overlap (i.e., values represented by group ‘abc’ are significantly different from group ‘de’ but not ‘cde’).

|  | 0 µg/mL | 1.56 µg/mL | 3.125 µg/mL | 6.25 µg/mL | 12.5 µg/mL | 25 µg/mL | 50 µg/mL | 100 µg/mL | 200 µg/mL |
| --- | --- | --- | --- | --- | --- | --- | --- | --- | --- |
| PMET 540 | 32.7 | 27.3 | 31.2 | 43.1 | 26.7 | 33.1 | 29.0 | 3.38 | 0.59 |
|  | abc | abcde | abcd | a | abcde | abc | abcde | de | e |
| PMET 731 | 30.9 | 48.2 | 43.0 | 41.9 | 34.5 | 30.3 | 13.8 | 11.3 | 3.97 |
|  | abcd | a | a | ab | abc | abcde | bcde | cde | de |
| PMET 885 | 39.9 | 28.3 | 24.6 | 29.7 | 28.4 | 24.7 | 29.6 | 32.5 | 21.8 |
|  | ab | abcde | abcde | abcde | abcde | abcde | abcde | abc | abcde |
